# Postoperative Pain Management Strategies Without Regional Analgesia in Knee Surgeries: A Scoping Review

**DOI:** 10.3390/medsci14010062

**Published:** 2026-01-30

**Authors:** Melissa Joo Young, Kevin Heebøll Nygaard, Gunhild Kjærgaard-Andersen, Christina Frøslev-Friis, Gayani Ranasinghe, Thomas Strøm, Rajesh Prabhakar Bhavsar

**Affiliations:** 1 Department of Anesthesiology and Intensive Care, University Hospital of Southern Denmark, 6200 Aabenraa, Denmark; melissa.joo.young@rsyd.dk (M.J.Y.); christina.froeslev-friis2@rsyd.dk (C.F.-F.);; 2 Department of Orthopedics, University Hospital of Southern Denmark, 6200 Aabenraa, Denmark; 3 Department of Regional Health Research, University of Southern Denmark, 6200 Aabenraa, Denmark; 4 Department of Intensive Care, Odense University Hospital, 5000 Odense, Denmark; thomas.stroem@rsyd.dk; 5 Department of Anesthesia and Intensive Care, University of Southern Denmark, 5000 Odense, Denmark

**Keywords:** knee surgery, postoperative, pain management, systemic analgesia

## Abstract

**Background/Objectives:** Intensive postoperative pain is a common challenge after knee surgeries such as total knee arthroplasty, arthroscopy, cruciate ligament or meniscus repair, and fixation of tibial plateau or distal femoral fractures. This scoping review mapped and summarized non-regional postoperative analgesia strategies to provide an overview of available approaches when regional blocks or neuraxial anesthesia are not feasible. Methods: We followed established methodological guidance for scoping reviews and report the data in accordance with the PRISMA-ScR checklist. We searched PubMed/MEDLINE, EMBASE, Scopus, and ClinicalTrials.gov in January 2025. Eligible designs included randomized controlled trials, non-randomized trials, observational studies, case series, and pilot studies. Results: We screened 3390 records and assessed 332 in full text. A total of 43 studies met the inclusion criteria, and the literature was grouped into: (1) arthroplasty, (2) arthroscopy, (3) cruciate ligament or meniscus repair, and (4) tibial plateau or distal femoral fractures. We identified substantial heterogeneity in interventions, comparators, and outcome measures across the first three sets of literature but found no focused articles for tibial plateau or distal femoral fractures. Most studies evaluated multimodal approaches combining systemic analgesics with local periarticular or intraarticular techniques. Evidence on functional recovery and mobilization was limited. Conclusions: Current evidence on non-regional postoperative analgesia in knee surgery is fragmented and varies considerably in design, intervention, and reported outcomes. Multimodal regimens and pre-emptive NSAID use were frequently associated with reduced early postoperative pain and lower opioid requirements, although comparability across studies remains limited. As existing evidence largely focuses on outcomes during hospitalization, future research should prioritize standardized pain and functional outcome reporting and directly compare systemic and local multimodal strategies, while extending follow-up beyond discharge to better characterize sustained clinical relevance.

## 1. Introduction

Knee surgeries such as total knee arthroplasty (TKA), knee arthroscopies, cruciate ligament or meniscus repair (CLMR), and fixation of tibial plateau or distal femoral fractures are standard procedures frequently associated with intense postoperative pain [1,2].

Severe postoperative pain complicates physiotherapy and early ambulation. Delayed mobilization increases length of stay (LOS) and healthcare costs [3,4]. Lack of early mobilization affects the surgical outcome because it increases the risk of joint stiffness, contractures, and muscle atrophy [5,6]. Insufficient pain management, high opioid consumption, and immobility caused by motor-blocking regional techniques may also increase the risk of adverse events such as deep venous thrombosis, pneumonia, and ileus [7,8,9]. In contrast, patients with well-managed postoperative pain have higher satisfaction scores, shorter hospital stays, improved early ambulation, and fewer postoperative complications [10,11].

Multimodal analgesic strategies have been introduced because they combine synergistic pharmacological effects with non-pharmacological mechanisms to improve pain control. These strategies include neuraxial anesthesia, regional nerve blocks, systemic analgesics, and local infiltration anesthesia [12]. Multimodal strategies may reduce opioid consumption and may limit adverse effects such as nausea, constipation, respiratory depression, and opioid-induced hyperalgesia [11,13,14,15]. Regional and neuraxial techniques are effective; however, the implementation of regional anesthesia is not resource-neutral, as it typically requires additional trained personnel, dedicated equipment (e.g., ultrasound), and non-trivial procedural time. In contemporary workflow analyses and service-implementation studies, these added inputs are explicitly highlighted (including the need for additional staffing/equipment and operating room time considerations), as, e.g., continuous peripheral nerve blocks have been reported to require approximately 10 min longer than single-injection blocks, with correspondingly higher estimated physician-time cost, thus also limiting their universal applicability [16,17]. In addition, side effects such as quadriceps weakness that may increase fall risk, are often seen in regional anesthesia [18]. As surgical techniques evolve, clinicians have explored non-regional strategies to provide adequate analgesia without relying on nerve blocks or spinal anesthesia.

Although multimodal pain management is widely used, the available literature is fragmented across procedures, drug classes, and study designs. No review has mapped non-regional postoperative strategies across the full range of knee surgeries. The absence of an overview makes it challenging to understand which systemic, local, or non-pharmacological strategies are available and where essential knowledge gaps remain. The literature also varies in age groups, functional demands, and rehabilitation trajectories, especially when comparing arthroplasty, arthroscopy, ligament reconstruction, and fracture surgery. These populations differ markedly in invasiveness and postoperative pain profiles, but all require adequate non-regional analgesia when regional techniques are not feasible.

This scoping review aimed to map the available non-regional postoperative analgesia strategies for knee surgery and to identify knowledge gaps that may inform future clinical trials and systematic reviews.

## 2. Materials and Methods

### 2.1. Study Design

We conducted a scoping review to systematically map and describe non-regional pain management strategies used in knee surgery. The review followed established methodological guidance and framework for scoping reviews from Arksey and O’Malley, and adhered to the PRISMA-ScR (Preferred Reporting Items for Systematic Reviews and Meta-analysis Guidelines Extension for Scoping Reviews). The protocol was developed a priori and registered at the Open Science Framework (https://doi.org/10.17605/OSF.IO/DVSRF (accessed on 24 December 2025)).

Given the exploratory nature of scoping reviews, heterogeneous surgical procedures were intentionally included to allow for comprehensive mapping of strategies potentially transferable across clinical contexts. Because the objective was descriptive rather than comparative, no formal meta-analysis was conducted.

The PCC framework (Table 1) was used to help define the research question and map the scope of this review.

Eligible study designs included randomized controlled trials, non-randomized trials, observational studies, case series, and pilot studies, with interventions initiated from admission for surgery until discharge. The focus of our review is perioperative pain management strategies, as many pain management strategies which influence postoperative pain include a combination of both pre-, intra- and postoperative interventions whose effects cannot be clearly distinguished from one another. Therefore, to preserve a broad study selection, we included studies with interventions starting from admission for surgery to postoperative discharge.

### 2.2. Exclusion Criteria

- Studies evaluating regional or neuraxial techniques, also in combination with non-regional pain management techniques.
- Surgical technique modifications, in which the surgical procedure itself was intentionally altered in a way likely to independently affect postoperative pain or functional recovery (e.g., minimally invasive or muscle-sparing approaches, navigation- or robotic-assisted techniques, altered tourniquet strategies, or the incorporation of periarticular local infiltration analgesia as part of the surgical technique rather than the anesthetic regimen).
- Non-eligible designs (editorials, letters, reviews, protocols).
- Studies reporting only long-term pain outcomes defined as time post-discharge.
- Studies including pediatric populations.

### 2.3. Information Sources and Search Strategy

We searched PubMed/MEDLINE, Embase, Scopus, and ClinicalTrials.gov in January 2025. Reference lists of included studies were screened for additional eligible literature.

Search strategies combined three core concepts: postoperative pain management, knee surgery, and exclusion of regional anesthesia. The review team developed the search strategy, which was externally peer-reviewed by an experienced medical librarian in accordance with the Peer Review of Electronic Search Strategies (PRESS) recommendations. Full search strings are provided in File S1.

### 2.4. Study Selection

All records were managed in Covidence, (Covidence systematic review software, Veritas Health Innovation, Melbourne, Australia. Available at: www.covidence.org, 26 January 2025) where duplicates were removed. Two reviewers independently screened titles and abstracts, followed by full-text screening of potentially eligible studies. Discrepancies were resolved by consensus or consultation with a third reviewer. Reasons for full-text exclusion were documented.

### 2.5. Data Extraction

Data from included studies were extracted using a pre-piloted form capturing study characteristics, population, surgical procedure, anesthesia technique, co-analgesics, intervention, comparator details, acute postoperative pain outcomes, and adverse events.

### 2.6. Data Synthesis

The extracted data were synthesized using a descriptive, iterative approach appropriate for scoping reviews. We first grouped studies according to the class of systemic analgesic intervention evaluated (e.g., opioids, acetaminophen, NSAIDs/COX-2 inhibitors, gabapentin, ketamine, intravenous lidocaine, alpha-2 agonists, corticosteroids, magnesium, nefopam, duloxetine). Within each class, findings were further stratified by surgery type (arthroplasty, arthroscopy, ligament reconstruction) and study design. Key outcome domains (pain intensity scores, opioid consumption, time to mobilization, length of stay, and adverse events) were tabulated to allow visual comparison across interventions and study types. Narrative synthesis was then used to summarize trends, highlight consistencies or discrepancies between studies, and identify evidence gaps. Meta-analysis was not planned a priori but was considered only if ≥3 sufficiently homogeneous RCTs were available for a specific intervention and outcome.

## 3. Results

**Figure 1:**
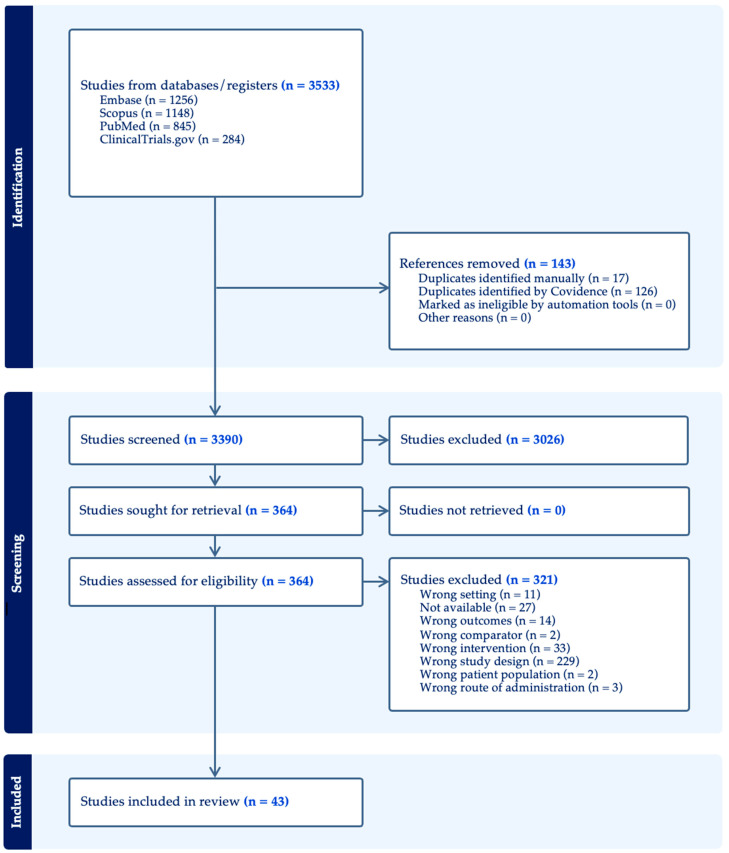
PRISMA 2020 flow diagram for study selection.

A total of 3533 records were identified through database searches (Embase = 1256; Scopus = 1148; PubMed = 845; ClinicalTrials.gov = 284). After removal of 143 duplicates (17 manually and 126 via Covidence), 3390 unique records remained for title and abstract screening. Of these, 3026 were excluded as clearly irrelevant. The remaining 364 full-text articles were assessed for eligibility, and 320 were subsequently excluded, primarily due to unsuitable study design (*n* = 229), wrong intervention *(n* = 33), unavailable full text (n = 27), wrong setting (*n* = 11), non-matching outcomes (*n* = 14), or other ineligibility criteria such as wrong comparator, population, or route of administration. No studies were excluded due to retrieval failure. Ultimately, 43 studies met the inclusion criteria and were included in this scoping review (Figure 1).

The included studies represented a geographically diverse sample, with the most considerable contributions from China (*n* = 9) and the United States (*n* = 6). European countries accounted for a substantial proportion of the evidence base, with France (*n* = 3), Belgium (*n* = 2), and Italy (*n* = 2) leading, and others with a single publication each. Only a few studies originated from the Middle East or Asia outside China, reflecting uneven global research activity (File S2).

These studies collectively examined a broad spectrum of non-regional analgesic and non-pharmacological strategies used in knee surgery, representing diverse surgical contexts, pharmacological regimens, and study designs. To facilitate comparison, the included studies were categorized into three surgical groups based on procedure type and clinical context: total knee arthroplasty (TKA), arthroscopic procedures and cruciate ligament or meniscus repair (CLMR). We found no focused literature on non-regional analgesic and non-pharmacological strategies used in tibial plateau or femoral fracture surgery, and no data was grouped for this category.

The following sections present detailed findings for each group, describing patient demographics, intervention types, mobilization outcomes, and patterns of multimodal pain management. These subgroup summaries are then synthesized for comparison to highlight overarching trends, knowledge gaps, and evolving clinical directions across all knee surgery populations.

### 3.1. Total Knee Arthroplasty (TKA) Set: Table 2

A total of seventeen studies, including 2243 participants, were analyzed. The mean of reported mean ages was approximately 62.0 years (median of means 65.1 years), sex was reported in 12 studies accounting for 1513 participants, and the average proportion of males was about 32%. Mobilization outcomes were assessed in 10 of the 17 studies, typically using range of motion (ROM), timed up-and-go (TUG), time-to-walk, or continuous passive motion (CPM) tolerance. However, many trials did not include functional endpoints. Interventions were predominantly pharmacological (12 studies), with two non-pharmacological strategies (continuous cold-flow therapy and music), two studies of local pharmacological approaches (periarticular infiltration or local infiltration anesthesia (LIA)), and one study comparing a mixed regimen (cryotherapy vs. IV tranexamic acid). Across the pharmacological studies, patient-controlled opioid analgesia (morphine, fentanyl, or sufentanil) was commonly employed, usually within a multimodal background of acetaminophen and/or NSAIDs, often supplemented with LIA. Several trials directly compared route or timing of administration, including IV versus oral acetaminophen, pre- versus postoperative NSAIDs, IV versus oral NSAIDs, and the addition of steroids to LIA.

**Table 2:** Characteristics and reported outcomes of studies evaluating perioperative pain management strategies in total knee arthroplasty (TKA).

Study	Country	Design	*n*	Mean Age	Intervention	Comparator	Pain Management System	Mobilization Assessed	Primary Outcome	Secondary Outcomes	Headline Results	Conclusion	Authors Suggestions
Motamed et al. 2000 [19]	France	Randomized, double-blind, placebo- and active-controlled	37		IV (pre-emptive morphine vs. M-6-G)	Placebo and active	Morphine PCA 24 h	No	Morphine consumption 24 h	Pain VAS; time to first PCA; AEs	Pre-emptive morphine ↓ early pain and 24 h morphine; M-6-G ineffective	Pre-emptive IV morphine beneficial; M-6-G not	Consider timing; avoid M-6-G
Wadhwa et al. 2001 [20]	Australia	Randomized, double-blind, placebo-controlled	66		Oral (dextromethorphan)	Placebo	Morphine PCA 24 h; CPM for 24 h	Yes (CPM tolerance)	24 h morphine use	Pain during CPM; side effects	↓ morphine ~29%; no pain benefit; ↑ nausea	Not clinically useful despite opioid sparing	Avoid routine dextromethorphan
Zippel & Wagenitz 2006 [21]	EU	Multicenter RCT, double-blind, equivalence	252	60	IV (dexketoprofen vs. ketoprofen)	Active comparator	Rescue morphine/paracetamol/propacetamol	No	SAPID 0–8 h (VAS)	PID; rescue use; AEs	Equivalent analgesia; trend fewer AEs with dexketoprofen	Either NSAID acceptable; tolerability may favor dexketoprofen	Consider dexketoprofen for tolerability
Sıvrıkoz et al. 2014 [22]	Turkey	Randomized, double-blind, placebo-controlled	118	60	IV (dexketoprofen vs. lornoxicam)	Placebo	Morphine PCA 24 h	No	VAS rest/move	Morphine use; PCA boluses	Both ↓ pain/opioids; dexketoprofen superior	Prefer dexketoprofen among tested NSAIDs	Use IV dexketoprofen 12-hourly
Chen et al. 2015 [23]	Taiwan	RCT	30	68	Music (audio)	Usual care	Standard GA; opioids PRN	No	Physiologic parameters; postop VAS	Opioid dose	BP stabilization; no pain/opioid difference	Music may modulate stress, not analgesia	Further research; feasible low-risk adjunct
Ban et al. 2017 [24]	China	Prospective randomized (single-blind)	110	64	Periarticular multimodal injection	Saline	Sufentanil PCA + celecoxib	Yes (ROM and activity)	NRS pain; NSAID use; WOMAC; LOS	Satisfaction; AEs	Lower pain; better ROM; shorter LOS	Periarticular injection improves early recovery	Adopt LIA routinely
Politi et al. 2017 [25]	USA	Prospective randomized	120		IV vs. PO (acetaminophen)	Route comparison	Multimodal; hydromorphone/oxycodone	No	24 h VAS; hydromorphone use	Early 0–4 h VAS; cost	Equivalent 24 h pain/opioids; small early benefit IV	Prefer PO due to cost; IV limited role	Use oral acetaminophen routinely
Hickman et al. 2018 [26]	USA	Randomized, double-blind, placebo-controlled equivalence	486		IV vs. Oral (acetaminophen)	Equivalence (IV vs. PO)	Standardized multimodal	Yes (time to walk 10 ft)	24 h opioid MME	Pain; PONV; PACU; LOS; time to ambulation	Oral equivalent to IV	Use oral as cost-effective	Prefer oral route
Xiao et al. 2018 [27]	China	Retrospective cohort	300	62.05.00	IV (flurbiprofen axetil) vs. Oral (celecoxib) vs. None	FA vs. CX vs. placebo	Morphine PCA	Yes (ROM)	VAS rest/move	Morphine use; ROM; LOS; AEs	FA best early pain/↓opioids; both shortened LOS	FA and CX beneficial; FA > CX early	Prefer FA when available; otherwise CX
Kim et al. 2019 [28]	South Korea	Prospective RCT, double-blind	67	66	IV (dexmedetomidine infusion)	Saline	Fentanyl PCA 48 h	No	Inflammation markers (IL-6, TNF-α)	Pain scores; PCA use; hemodynamics	Lower pain at 12–48 h; no opioid/LOS benefit	Dexo reduces pain/inflammation without opioid sparing	Potential adjunct for pain; monitor HR
Shao et al. 2020 [29]	China	RCT	196	68.05.00	Oral (meloxicam)	Preop vs. postop timing	Fentanyl+tramadol PCA 48 h	Yes (HSS at 3 mo)	VAS rest/flexion; PCA use	PGA; HSS 3 mo; AEs	Preop dosing ↓ early pain and PCA use	Prefer preop meloxicam timing	Administer meloxicam preop
Peng et al. 2021 [30]	China	Within-patient RCT (bilateral TKA)	60	65.01.00	Periarticular cocktail ± steroid	Cocktail without steroid	Morphine PCA 3 days; NSAIDs	Yes (ROM; HSS)	VAS rest/move	ROM; swelling; temp; preference; AEs	No added benefit from steroid	Steroid unnecessary in robust LIA	Use LIA without steroid routinely
Coviello et al. 2022 [31]	Italy	Prospective randomized case–control	100	71	Device (continuous cold flow)	Gel ice packs	Standard multimodal; tramadol rescue	Yes (ROM)	VAS pain (T0–day5)	Tramadol use; ROM; transfusions; satisfaction	Lower 24 h VAS and less tramadol; better ROM early	CCF safer/more effective than gel packs for early recovery	Adopt CCF for pain control after TKA
Yuan et al. 2022 [32]	China	Prospective RCT, double-blind	100	68	Oral (duloxetine)	Placebo	Multimodal + LIA + PCA rescue	Yes (ROM)	rVAS and aVAS over 2 w	Opioid use; ROM; AEs	Lower pain; ↓ opioids; better early ROM; fewer N/V/constipation	Duloxetine effective adjunct	Add perioperative duloxetine selectively
Paulin et al. 2023 [33]	India	Double-blind RCT	49	22	IV (ketamine bolus+infusion)	Saline	Morphine PCA 72 h	Yes (TUG, ROM)	TUG at 72 h	Pain; morphine use; ROM; discharge day; OKS	No significant improvement in TUG, pain, or opioids	Low-dose ketamine not beneficial in this setting	Avoid routine ketamine for oncologic TKA
Kraus et al. 2024 [34]	USA	RCT	76	66	Genetics-guided prescribing (preop test)	Standard care	Multimodal incl. periarticular local anesthetic	No	OBAS at 24 h	VAS; opioid use; side effects; satisfaction	No benefit on pain or opioids	Routine pharmacogenomics not useful in standard-risk TKA	Do not adopt routinely for pain control
Sönmez Sağlam et al. 2025 [35]	Türkiye	Prospective RCT (3-arm)	76	65	Cryotherapy device; IV tranexamic acid	Gel cold packs	Diclofenac IM + paracetamol IV	Yes (ROM)	Blood loss; VAS	ROM; complications	Cryo ↓ pain and preserved ROM; TXA ↓ blood loss	Both strategies enhance recovery (different domains)	Consider cryotherapy; use TXA for blood-sparing

This table summarizes clinical studies assessing pharmacological, device-based, and non-pharmacological interventions for postoperative pain control following total knee arthroplasty. Studies are presented chronologically and include information on study design, population characteristics, interventions and comparators, pain management protocols, mobilization strategies, primary and secondary outcomes, headline results, and authors’ conclusions and recommendations. Abbreviations: AE, adverse event; aVAS, activity visual analogue scale; BP, blood pressure; CCF, continuous cold flow; CPM, continuous passive motion; CX, celecoxib; FA, flurbiprofen axetil; GA, general anesthesia; HSS, Hospital for Special Surgery knee score; IV, intravenous; LIA, local infiltration analgesia; LOS, length of stay; MME, morphine milligram equivalents; NRS, numeric rating scale; NSAID, non-steroidal anti-inflammatory drug; OBAS, Overall Benefit of Analgesia Score; OKS, Oxford Knee Score; PCA, patient-controlled analgesia; PID, pain intensity difference; PONV, postoperative nausea and vomiting; PRN, as needed; ROM, range of motion; rVAS, rest visual analogue scale; SAPID (SPID), summed analgesic pain intensity difference; TKA, total knee arthroplasty; TUG, Timed Up and Go test; TXA, tranexamic acid; VAS, visual analogue scale; WOMAC, Western Ontario and McMaster Universities Osteoarthritis Index; ↓, decreased/lowered; ↑, increased.

### 3.2. Arthroscopy Set: Table 3

Eighteen studies with 1870 participants were included. The reported mean age was approximately 42.0 years (median of means = 43 years), with an average of ~50% male participants across the 7 studies that reported sex distribution, accounting for 862 of the participants. Mobilization outcomes were assessed in only 2 studies, with assessments limited to range of motion or day-to-day activities in a few trials. Interventions were dominated by pharmacological strategies, including systemic NSAIDs, opioids, and a wide range of intra-articular adjuvants, while non-pharmacological strategies were almost absent. Routes of administration spanned oral (celecoxib, loxoprofen, meloxicam, oxycodone/acetaminophen, zolpidem), intravenous (magnesium, NSAIDs), and intra-articular (ketamine, clonidine, neostigmine, levobupivacaine, sufentanil, warmed lidocaine).

**Table 3:** Characteristics and reported outcomes of studies evaluating perioperative pain management strategies in arthroscopic knee surgery.

Study	Country	Design	*n*	Mean Age	Procedure	Intervention	Comparator	Pain Management System	Mobilization Assessed	Primary Outcome	Secondary Outcomes	Headline Results	Conclusion	Authors Suggestions
Koinig et al. 1998 [36]	Austria	Randomized double-blind placebo-controlled	46		Diagnostic/therapeutic AKS under TIVA	IV magnesium sulfate infusion	IV placebo	Propofol + fentanyl TIVA; postop fentanyl as needed	No	Intra- and postoperative fentanyl use	VAS 5–240 min; AEs	↓ intra/postop fentanyl; VAS similar	Opioid-sparing without VAS change	Consider Mg for opioid reduction
Reuben & Connelly 1999 [37]	USA	Randomized double-blind (5-arm)	50		Outpatient meniscal arthroscopy	IA bupivacaine ± clonidine (or clonidine alone); SC clonidine variants	Multiple active comparators including IA saline group equivalence	Oral codeine–acetaminophen (Tylenol 3)	No	Time to first analgesic (duration)	VAS; opioid tablets; discharge time	IA bupivacaine + clonidine prolonged duration; fewer tablets	Add IA clonidine to bupivacaine	Use IA clonidine 1 µg/kg with bupivacaine
Gentili et al. 2001 [38]	France	Randomized, double-blind, 6-arm	84		Meniscus repair (ambulatory)	IA clonidine and/or IA neostigmine (± SC counterpart)	IA + SC saline	Rescue paracetamol 1 g PO (no opioids)	No (but VAS during knee flexion)	VAS rest and mobilization 1–24 h	Time to first paracetamol; total doses; AEs	All active IA regimens ↓ VAS vs. placebo; clonidine ↑ hypo/brady	IA clonidine/neostigmine effective; combination not superior	Choose agent mindful of clonidine hypotension
Vranken et al. 2001 [39]	Netherlands/Belgium	Randomized double-blind (3-arm)	60	37	Diagnostic day-case AKS	IA sufentanil (5 or 10 µg) vs. IV 5 µg	IV sufentanil	Paracetamol intraop; PO paracetamol postop	No	VAS T1–T4	Paracetamol use; time to PACU discharge	IA sufentanil ↓ pain vs. IV; faster discharge eligibility	IA sufentanil 5 µg effective and safe	Prefer IA over IV sufentanil in day-case AKS
Ménigaux et al. 2005 [40]	France	Randomized, double-blind, placebo-controlled	40	32	ACL repair (arthroscopic)	Oral (gabapentin)	Placebo	GA + PCA morphine 48 h; ketoprofen PRN	Yes (ROM days 1–2)	VAS pain; 0–48 h morphine	Anxiety VAS; AEs; early ROM	↓ PACU pain; ↓ 48 h morphine by ~58%; ↑ ROM day 1–2	Gabapentin enhances early recovery and opioid-sparing	Consider preop gabapentin 1200 mg
Arai et al. 2006 [41]	Japan	Randomized controlled (single-blind)	24	64	Partial meniscectomy	IA warmed lidocaine vs. room-temp	Temperature comparison	Pentazocine premed; diclofenac PRN	No	Intraoperative pain VAS	Intraop pentazocine; postop diclofenac	Warmed lidocaine ↓ intraop pain and analgesic use	Temperature optimization improves IA local anesthetic effect	Warm IA lidocaine to ~40 °C
Jacobson et al. 2006 [42]	Sweden	Prospective randomized (NSAID vs. coxib)	122	48	Outpatient AKS (meniscus/synovium/diagnostic)	Oral lornoxicam vs. rofecoxib	Coxib vs. NSAID	Paracetamol PACU; IA fentanyl + local portal infiltration; rescue dextropropoxyphene	No	Rescue need days 0–4	VAS; side effects; satisfaction	No difference; many still need rescue	Conventional NSAID reasonable first choice	No added benefit of coxib in this setting
Tashjian et al. 2006 [43]	USA	Randomized, double-blind (zolpidem vs. placebo) + open standard-care arm	68	47	Outpatient meniscus/loose bodies	Oral (zolpidem nights 1–7)	Placebo and standard care	Hydrocodone/acetaminophen + ibuprofen; standard rehab	No	Daily VAS pain 7 d; opioid tablets used	Fatigue VAS; AEs	↓ pain and opioid tablets with zolpidem; ↓ fatigue	Improved sleep lowers pain/opioid use first week	Consider short zolpidem course post-arthroscopy
Hashemi et al. 2010 [44]	Likely Iran	Randomized, double-blind (3-arm)			AKS (general)	IA pethidine (preemptive vs. preventive)	IA saline	Rescue systemic morphine PRN	No	VAS 1–24 h	Time to first morphine; 24 h morphine	Preemptive dosing best: ↓ VAS, ↓ morphine, delayed first dose	Timing matters; preemptive IA pethidine superior	If using IA opioid, give pre-incision
Sun et al. 2013 [45]	China	Systematic review/meta-analysis of 7 RCTs	230		Arthroscopic knee surgery (mixed)	Intraarticular clonidine	IA placebo	Standard GA across RCTs	No	VAS 1–24 h	Rescue need; AEs (nausea ↓; hypotension ↑)	Small, short-lived VAS reduction ≤4 h; ↓ rescue; ↑ hypotension risk	Adjunct only; monitor BP	IA clonidine not standalone; consider as add-on
Isik et al. 2014 [46]	Türkiye	Randomized, double-blind, placebo-controlled (3-arm)	60		Partial meniscectomy	IA ketamine ± levobupivacaine	IA saline	Rescue tramadol IV as needed	No	VAS at 1–24 h	Rescue tramadol; side effects	IA ketamine improves early pain; ketamine+levobupivacaine best at 4–12 h; ↓ early tramadol	Adding local anesthetic augments IA ketamine effect	Consider IA ketamine + levobupivacaine
Onda et al. 2016 [47]	Japan	Randomized parallel-group (3-arm)	160		Second-look after ACL or meniscal procedures	Oral (celecoxib vs. loxoprofen vs. acetaminophen)	Three-way comparison	No routine opioids; pre-discharge IV flurbiprofen; rebamipide prophylaxis	Yes (daily activities allowed POD1 per pain)	VAS rest/movement to 48 h	Subjective global relief; AEs	Celecoxib < acetaminophen at 24–48 h; loxoprofen < acetaminophen at 48 h rest	COX-2/NSAID superior to acetaminophen early	Prefer celecoxib for AKS acute pain
Zhou et al. 2017 [48]	China	Randomized, open-label (3-arm)	182	36	Meniscal arthroscopy	Oral celecoxib (−24 h vs. −1 h vs. +4 h)	Timing arms	Rescue pethidine 5 mg/kg	No	VAS at rest and 90° flexion 12–24 h	PGA; rescue use; AEs	Preop (−24 h/−1 h) < postop (+4 h) for pain; rescue trend ↓	Preemptive celecoxib effective; −1 h optimal	Dose celecoxib preop (preferably −1 h)
Uribe et al. 2018 [49]	USA	Randomized double-blind active-comparator pilot	51	43,7	Outpatient AKS (mixed)	IV ibuprofen (preop) vs. IV ketorolac (postop)	Active comparator	Standard GA; PACU hydromorphone rescue; home ibuprofen + oxy/APAP	No	PACU VAS	PACU opioids; 24 h opioids; time to first opioid	Preop ibuprofen ↓ arrival VAS & PACU opioids; effect fades by 2 h	Preemptive IV NSAID helpful for very early period	Use IV NSAID preop if IV route planned
El Baz & Farahat 2019 [50]	Egypt	Randomized double-blind (3-arm)	90	27,5	Diagnostic/therapeutic AKS	IA levobupivacaine ± dexmedetomidine	IA saline	Rescue meperidine IV	No	VAS 0.5–24 h	Time to first rescue; total meperidine; AEs	Adding dexmedetomidine ↓ VAS and opioids markedly	α2 augmentation of IA local anesthetic is effective	Consider IA dexmedetomidine adjuvant
Hou et al. 2019 [51]	China	Randomized, non-blinded	296	37,5	AKS (mixed procedures)	Oral meloxicam (pre vs. post)	Postoperative dosing	Rescue pethidine	No (3-mo function only)	VAS rest/flexion; PGA	Rescue pethidine; ROM/IKDC/Lysholm 3 mo	Preop dosing ↓ early (≤12 h) pain & PGA; ↓ rescue	Preemptive meloxicam beneficial early	Give meloxicam preoperatively
Sağır et al. 2020 [52]	Türkiye	Randomized, double-blind (3-arm)	75	46,7	Arthroscopic meniscectomy	Intraarticular ketamine (0.5 vs. 1 mg/kg) + periarticular bupivacaine	Intraarticular saline + bupivacaine	PCA morphine in PACU; GA with fentanyl	No	VAS rest/move; time to first analgesic	Morphine use; discharge time; AEs	1 mg/kg reduced early morphine; no 24 h total or discharge time effect	IA ketamine provides early opioid-sparing only	If used, 1 mg/kg offers best early benefit
Liu et al. 2021 [53]	China	Randomized, controlled (parallel)	232	43	AKS (mixed procedures)	Oral oxycodone/acetaminophen vs. oral celecoxib	Celecoxib	Rescue pethidine PRN	No	VAS rest/motion 6–72 h	Rescue use; satisfaction; AEs	OPT better ≤24 h; ↓ rescue pethidine; similar AEs	OPT superior for early pain	Prefer OPT when regional not used

This table summarizes randomized trials, observational studies, and systematic reviews evaluating pharmacological and intra-articular interventions for perioperative pain management in arthroscopic knee surgery. Included studies span diagnostic and therapeutic procedures and report on study design, patient characteristics, type and timing of interventions, comparators, perioperative analgesic regimens, mobilization assessment, primary and secondary outcomes, headline results, and authors’ conclusions and recommendations. Abbreviations: AE, adverse event; AKS, arthroscopic knee surgery; APAP, acetaminophen; ACL, anterior cruciate ligament; COX-2, cyclooxygenase-2 inhibitor; GA, general anesthesia; IA, intra-articular; IKDC, International Knee Documentation Committee score; IV, intravenous; NSAID, non-steroidal anti-inflammatory drug; PACU, post-anaesthesia care unit; PCA, patient-controlled analgesia; PGA, patient global assessment; POD, postoperative day; PO, oral administration; PRN, as needed; ROM, range of motion; SC, subcutaneous; TIVA, total intravenous anaesthesia; VAS, visual analogue scale; ↓, decreased/lowered; ↑, increased.

### 3.3. Cruciate Ligament or Meniscus Repair (CLMR) Set: Table 4

Eight studies with 1722 participants were included. The reported mean age was approximately 33.5 years (median of means ~32.1 years), with an average of ~63% male participants in the 7 studies that reported sex, accounting for 1006 of the included participants. Mobilization or physiotherapy outcomes were only reported in one cohort, noting rehabilitation beginning on postoperative day two and no quantitative mobilization outcomes reported. The interventions tested covered a range of systemic and local strategies, including systemic NSAID timing comparisons, systemic drug combinations, intra-articular adjuvants (remifentanil, dexmedetomidine, sufentanil), and non-pharmacologic intraoperative irrigation protocols.

**Table 4:** Characteristics and reported outcomes of studies evaluating perioperative pain management strategies in cruciate ligament or meniscus repair surgery (CLMR).

Study	Country	Design	*n*	Mean Age	Procedure	Intervention	Comparator	Pain Management System	Mobilization Assessed	Primary Outcome	Secondary Outcomes	Headline Results	Conclusion	Authors Suggestions
Barber & Gladu 1998 [54]	USA	Double-blind randomized multicenter	125	29	ACLR (patellar-tendon autograft)	Oral ketorolac vs. hydrocodone + APAP (after parenteral loading for ketorolac)	Opioid combo	Uniform GA; CPM; local infiltration	No (CPM used uniformly)	SPID/TOTPAR at 3–4 h post dose	Global ratings; AEs	In outpatients (early dosing) ketorolac > hydrocodone+APAP	NSAID strategy effective early with loading dose	Use oral ketorolac post-ACLR with prior parenteral dose
Al-Metwalli et al. 2008 [55]	Saudi Arabia	Randomized, double-blind, placebo-controlled	60	38	Partial meniscectomy	IA dexmedetomidine 1 µg/kg vs. IV vs. placebo	IV dEX; IA saline	Diclofenac IV primary; tramadol PRN	No	VAS 1–24 h	Time to first analgesic; diclofenac use; haemodynamics; sedation	IA dEX ↓ VAS up to 6 h; delayed first analgesic; ↓ diclofenac; fewer systemic effects vs. IV	IA dexmedetomidine effective and safe adjunct	Prefer IA over IV dEX for AKS
Armellin et al. 2008 [56]	Italy	Randomized, double-blind controlled	120	30	Outpatient ACL reconstruction	IA ropivacaine + clonidine ± sufentanil 5 µg	Without sufentanil	Preemptive ketorolac; postop APAP+ketoprofen; cryotherapy	No	VAS 1–24 h	Rescue sufentanil; discharge timing; AEs	Pain low both; fewer rescues with IA sufentanil in 1st hour	Opioid add-on not necessary with robust LIA	Ropivacaine + clonidine sufficient for routine cases
Elseify et al. 2011 [57]	Qatar/Egypt	Randomized, double-blind, 3-arm	60	31.5	Primary ACL reconstruction	IV paracetamol vs. IV parecoxib vs. both	Active comparators	Rescue IV morphine to VAS ≤3	No	VAS rest/movement (PACU, 2 h, 8 h)	Morphine totals to 8 h; PACU time; satisfaction	Combo ↓ movement pain early; ↓ morphine; shorter PACU time	Paracetamol+parecoxib best for early ACLR pain	Combine IV paracetamol + parecoxib
Mardani-Kivi et al. 2013 [58]	Iran	Triple-blind randomized placebo-controlled	117	26	ACLR or partial meniscectomy	Celecoxib 400 mg PO −2 h (single dose)	Placebo	Rescue pethidine 0.5 mg/kg IV	No	VAS 6 h and 24 h	Pethidine consumption; AEs	Celecoxib ↓ VAS and pethidine at 6 and 24 h	Effective preemptive COX-2 for AKS	Use celecoxib 400 mg preop
Ma et al. 2021 [59]	China (multicentre)	Randomized controlled trial	464	41	AKS (ligament recon, meniscectomy, synovectomy, IAFR)	Preop NSAIDs (celecoxib/meloxicam/rofecoxib) vs. postop	Preop vs. postop dosing	Standard GA; rescue pethidine	No	VAS rest and passive movement	Rescue pethidine; satisfaction; AEs	Preop dosing ↓ early (≤24 h) pain and rescue opioids	Preemptive NSAIDs superior early	Give NSAID ~2 h preop
Wang et al. 2021 [60]	China/ South Korea	Retrospective comparative cohort	716	40	Single-bundle ACL reconstruction (non-acute)	No irrigation vs. 1 L vs. 3 L; 23 °C vs. 36 °C vs. 4 °C	Different irrigation volume/temperature	NSAIDs, cold compresses; early rehab from POD2	Yes (rehab started POD2; no quantitative endpoint)	VAS pain; swelling; skin temperature	CRP, IL-1/6/10	No differences in pain, swelling, or inflammatory markers	Irrigation volume/temperature had no clinical effect	Standardize; choose based on practicality
Alipour et al. 2023 [61]	Iran	Randomized, double-blind, placebo-controlled	60	32.7	Meniscectomy (*n* = 44) and ACL reconstruction (*n* = 16)	Intraarticular remifentanil 200 µg	IA saline placebo	GA (propofol/sevo); postop meperidine 25 mg if VAS ≥4	No	VAS 1–24 h	Analgesic requests, time to first dose, total meperidine	Large ↓ in VAS at all times; median 0 mg meperidine vs. 100 mg control	IA remifentanil markedly reduces pain and opioid use	Consider IA remifentanil (cartilage safety to consider)

This table summarizes randomized and observational studies evaluating perioperative analgesic strategies in patients undergoing cruciate ligament reconstruction or meniscus repair surgery. Studies are presented with details on study design, patient demographics, surgical procedure, type and timing of analgesic interventions, comparators, perioperative pain management systems, assessment of mobilization, primary and secondary outcomes, headline results, and authors’ conclusions and clinical recommendations. Abbreviations: ACL, anterior cruciate ligament; ACLR, anterior cruciate ligament reconstruction; AE, adverse event; AKS, arthroscopic knee surgery; APAP, acetaminophen; COX-2, cyclooxygenase-2 inhibitor; CPM, continuous passive motion; CRP, C-reactive protein; dEX, dexmedetomidine; GA, general anesthesia; IA, intra-articular; IAFR, intra-articular fracture repair; IL, interleukin; IV, intravenous; NSAID, non-steroidal anti-inflammatory drug; PACU, post-anaesthesia care unit; POD, postoperative day; PO, oral administration; PRN, as needed; SPID, summed pain intensity difference; TOTPAR, total pain relief; VAS, visual analogue scale; ↓, decreased/lowered; ↑, increased.

### 3.4. Comparison Across Groups

Seventeen studies with a total of 2243 participants were included in the TKA studies and involved the oldest populations (mean ~62 years, ~32% male) and more often assessed mobilization outcomes (10/17, typically ROM, TUG, or time-to-walk), with interventions spanning from pharmacological, local periarticular infiltration to non-pharmacologic, and mixed strategies. Arthroscopy studies (18 trials, 1870 participants) focused on a younger cohort (~42 years) more balanced in sex (~50% male), with mobilization rarely assessed and outcomes limited to very early pain (0–24 h) and day-case recovery. The CLMR set (8 trials, 1722 participants) represented the youngest groups (~33.5 years, ~63% male), with mobilization outcomes almost absent, and tested both systemic combinations and potent intra-articular adjuvants.

Across all three surgical groups, the most frequently described intervention was multimodal pain management strategies comprising combining systemic non-opioid analgesics with LIA. Opioids were primarily used as rescue therapy in cases of postoperative pain. NSAIDs and COX-2 inhibitors were the most frequently reported systemic interventions in all three groups, often in combination with paracetamol. Local strategies such as periarticular injections and cryotherapy were widely represented in the TKA set, as with LIA with or without adjuvants (e.g., clonidine, dexmedetomidine, ketamine, opioids) in the arthroscopy and CLMR set. A broad range of adjunctive interventions was explored across all three groups, including α2-agonists, NMDA antagonists, antidepressants, sleep or stress-modulating therapies, with highly heterogeneous outcome measures. Overall, the literature mapped in this scoping review demonstrates a tendency to focus on multimodal, opioid-sparing analgesic strategies, with noticeable variation in intervention type and reported outcome measures depending on surgical procedure.

## 4. Discussion

This scoping review provides a comprehensive overview of current non-regional strategies for postoperative pain management in knee surgeries, which predominantly include TKA, arthroscopy, and CLMR. The strategies reflect a broad clinical shift toward multimodal regimens and reduced reliance on opioids, although the extent and composition of these approaches varied considerably across studies. The included studies revealed significant gaps in consistency, comparability, and limited evaluation beyond the early postoperative period, that require further investigation before a standardized regimen can be recommended. As a scoping review, the findings should be interpreted as a descriptive overview of current practices and evidence gaps rather than conclusions regarding comparative efficacy.

The most consistent observation is the central role of systemic multimodal pharmacotherapy. Regular administration of non-opioid agents, particularly NSAIDs and acetaminophen, was frequently reported to be associated with lower early postoperative pain scores or reduced opioid use, although reporting practices differed markedly. Some compared oral and intravenous formulations; others examined pre-emptive versus postoperative initiation. None of these comparisons established a clear superiority of one regimen.

The observed variation in the selection of agents, routes, and timing likely reflects both the complexity of postoperative pain mechanisms and the diversity of clinical settings in which knee surgery is performed [62].

Postoperative pain after knee surgery arises from multiple nociceptive sources, and no single drug can target all mechanisms [11]. Consequently, clinicians began to combine agents with complementary mechanisms to achieve additive or synergistic analgesia, while reducing opioid exposure and its related adverse effects [63]. Similarly, some investigators emphasized pre-emptive dosing to inhibit peripheral sensitization before tissue injury, whereas others prioritized postoperative initiation to align with the recovery workflow and minimize bleeding risk. Such variation also reflects the heterogeneity of patient populations: elderly arthroplasty recipients often have comorbidities limiting NSAID use, whereas younger sports patients can tolerate more aggressive anti-inflammatory therapy. This variation underscores the descriptive nature of the present review, as differences in pharmacological rationale, clinical pathways, and institutional resources preclude direct comparisons across studies. Differences in institutional analgesic policies, anesthetic practices, and the availability of adjunct modalities have further contributed to inconsistent regimens [64,65]. In essence, the multiplicity of systemic strategies represents a pragmatic clinical response to the complex and evolving challenge of balancing analgesic efficacy, opioid minimization, and safety across diverse patient groups. Local multimodal techniques offer another promising dimension. Peri-articular infiltration after TKA and intra-articular injection during arthroscopy or ligament reconstruction can deliver high drug concentrations at the surgical site while minimizing systemic exposure. The principle is well established, but the practical implementation varies widely. The included trials used numerous combinations of local anesthetics, alone or mixed with ketorolac, corticosteroids, morphine, or α_2_-agonists, applied in different volumes and at different tissue planes. Several studies have reported modest early analgesic effects, but consensus on the optimal composition and safety remains limited. Concerns about potential chondrotoxicity, delayed wound healing, and systemic absorption remain insufficiently studied [66]. To achieve comparability, future trials should adopt standardized infiltration mixtures, clearly define administration techniques, and include follow-up long enough to detect late complications. The goal should be to identify one or two reproducible, safe protocols that could be applied across knee procedures with minor adaptations.

Despite growing interest in multimodal regimens, non-pharmacological interventions remain notably under-represented in the literature. Only two TKA studies evaluated strategies such as music and continuous cold-flow therapy, while no trial addressed behavioral preparation, structured physiotherapy, or sleep optimization. This omission is striking, considering that enhanced recovery after surgery (ERAS) pathways emphasize multimodal, non-pharmacologic support to accelerate rehabilitation and reduce opioid need [67]. Non-drug measures can influence both the sensory and emotional components of pain and often improve patient satisfaction [68]. They are inexpensive, scalable, and rarely associated with harm [69]. Their limited inclusion suggests a persistent pharmacologic bias in trial design. These gaps highlight an opportunity for future trials to integrate non-pharmacological components more systematically and assess their additive value within multimodal pathways. Cold therapy, guided early mobilization, and patient education on realistic pain expectations could easily be standardized and tested across all knee procedures.

An important consideration is the evolving role of motor-sparing regional techniques in knee surgery, including adductor canal, iPACK, and genicular nerve blocks which have reduced the functional limitations traditionally associated with motor blockade in regional anesthesia [70]. These techniques, however, are time-limited, operator-dependent, and variably available, and they do not fully address central sensitization, inflammatory responses, or cognitive–emotional contributors to postoperative pain. In this context, non-regional analgesic strategies should be viewed as complementary rather than competitive, providing a scalable and broadly applicable foundation that ensures continuity of analgesia beyond block resolution. Notably, pain science education and expectation management were almost entirely absent from the included studies, despite their potential to improve pain perception, opioid use, and patient satisfaction [71]. Future research should therefore evaluate integrated perioperative approaches that combine non-regional multimodal analgesia, selective motor-sparing regional techniques when available, non-pharmacological interventions, and standardized functional outcome measures.

Another critical limitation identified in this review concerns the narrow scope of reported outcomes. Most trials assessed pain only within the first 24 h, typically using visual analogue scales at rest, with limited attention to pain during movement or rehabilitation. Functional such as range of motion, timed up-and-go, and time to independent walking, were primarily reported in TKA studies. They were rarely included in arthroscopy or CLMR trials (see File S3). The absence of standardized functional outcome domains further complicates cross-study interpretation, as measures differed in timing, definition, and clinical relevance. For younger and more active populations undergoing reconstructive procedures, functional recovery is a critical endpoint that has often been underrepresented. Adoption of standardized core outcome domains encompassing both pain and function would improve interpretability and clinical relevance. Incorporating measures such as quality of recovery, patient satisfaction, and readiness for rehabilitation would further ensure that postoperative pain research reflects outcomes meaningful to both clinicians and patients. Future research should move beyond isolated demonstrations of short-term analgesic benefit and instead focus on optimizing timing, duration, and patient selection while evaluating safety and functional recovery in parallel.

The heterogeneity observed across studies spanning differences in age groups, sex distribution, comorbidities, dosing regimens, and outcome measures highlights a persistent gap between controlled research settings and real-world clinical practice. Single-center studies, while valuable for of concept, often reflect the resources, routines, and patient populations of their institutions and therefore cannot be generalized [72]. Analgesic regimens are strongly influenced by local logistics, drug availability, staffing patterns, and departmental culture, which determine what is feasible rather than what is ideal [73]. Even when multicenter protocols exist, adherence may vary due to institutional policies on opioid prescribing, perioperative monitoring, or nursing workload [74]. Consequently, consensus guidelines and standardized pathways, though scientifically sound, often face practical barriers at the implementation level. Collaborative multicenter trials should preserve core methodological uniformity while allowing limited flexibility for local adaptation. Such pragmatism may partly explain the continued exploration of similar analgesic strategies across diverse settings.

Although most trials reported favorable short-term outcomes, the persistent proliferation of studies across different drug classes and modalities appears driven more by contextual and pragmatic motivations than by conflicting evidence. Variations in institutional practice and logistical constraints have repeatedly prompted investigators to test similar concepts in new configurations. This pattern reflects an ongoing effort to balance mechanistic rationale with real-world applicability, rather than genuine uncertainty regarding the efficacy of multimodal analgesic principles (Detailed study-level rationales and contextual motivations are summarized in File S4).

When the findings across systemic, local, and non-pharmacological domains are considered collectively, the evidence suggests a pragmatic rather than uniform approach to postoperative pain management in knee surgery. Systemic non-opioid therapy forms a consistent and evidence-based foundation, while local infiltration and non-pharmacological strategies provide variable but potentially additive benefits. However, the absence of standardized protocols, inconsistent reporting of functional outcomes, and methodological diversity across studies limit the ability to define a single optimal regimen applicable to all surgical contexts. Instead, the emerging consensus favors flexible multimodal combinations tailored to institutional logistics, patient characteristics, and surgical complexity. Such flexibility aligns with the descriptive purpose of this review and highlights the need for future standardized, comparative studies across knee surgery populations.

## 5. Conclusions

The evidence on non-regional postoperative analgesia in knee surgery is heterogeneous and unevenly distributed across procedures and study designs. Multimodal systemic strategies and pre-emptive NSAID use were frequently associated with lower early postoperative pain scores or reduced opioid consumption, although substantial variation in interventions and outcome reporting limits firm conclusions. Periarticular and intra-articular techniques were commonly applied as adjuncts to systemic regimens, reflecting their pragmatic role within multimodal pathways. As this scoping review focused on early postoperative outcomes, future research should adopt standardized pain and functional outcome measures and directly compare systemic and local multimodal approaches, while extending follow-up beyond the immediate postoperative period to better characterize sustained clinical relevance.

## Figures and Tables

**Table 1:** Definition of research question using PCC framework.

Review question	What non-regional anesthesia strategies are used for postoperative pain management in knee surgery?
Population	Adults > 18 years of age undergoing knee surgery *
Concept	Use of systemic analgesic strategies ^†^
Context	From peri- and postoperative periods across all knee surgery settings until postoperative discharge

* TKA/TKR, UKA, knee arthroscopy, anterior cruciate ligament/posterior cruciate ligament reconstruction, multi-ligament reconstruction, patellar surgery, tibial plateau or distal femur fracture fixation.^†^ Non-regional postoperative analgesia strategies, including systemic analgesics (opioids, acetaminophen, NSAIDs/COX-2 inhibitors, gabapentinoids, ketamine, IV lidocaine, alpha-2 agonists, corticosteroids, magnesium, nefopam, duloxetine), and local or intra-articular modalities, when considered part of a multimodal or systemic strategy or clinically relevant to mapping non-regional options.

## Data Availability

No new data were created for this review.

## Supplementary Materials

The following supporting information can be downloaded at: https://www.mdpi.com/article/10.3390/medsci14010062/s1, File S1: search strategy; File S2: figures and tables; File S3: distribution of outcome domains; File S4: overview of rational for included studies.

## Abbreviations

The following abbreviations are used in this manuscript:

ACL	anterior cruciate ligament
AKS	Arthroscopic knee surgery
CLMR	cruciate ligament or meniscus repair
CPM	continuous passive motion
GA	general anesthesia
IV	intravenous
LIA	local infiltration anesthesia
LOS	length of stay
NSAIDs	non-steroid anti-inflammatory drugs
PCL	posterior cruciate ligament
ROM	range of motion
SA	spinal anesthesia
TKA	total knee arthroplasty
TUG	timed up-and-go
UKA	unicompartmental knee arthroplasty
PRESS	Peer Review of Electronic Search Strategies

## References

[1] Grosu I., Lavand’homme P., Thienpont E. (2014). Pain after knee arthroplasty: An unresolved issue. Knee Surg. Sports Traumatol. Arthrosc..

[2] Price A.J., Alvand A., Troelsen A., Katz J.N., Hooper G., Gray A., Carr A., Beard D. (2018). Knee replacement. Lancet.

[3] Wainwright T.W., Gill M., A McDonald D., Middleton R.G., Reed M., Sahota O., Yates P., Ljungqvist O. (2020). Consensus statement for perioperative care in total hip replacement and total knee replacement surgery: Enhanced Recovery After Surgery (ERAS^®^) Society recommendations. Acta Orthop..

[4] Gan T.J. (2017). Poorly controlled postoperative pain: Prevalence, consequences, and prevention. J. Pain Res..

[5] Rhamelani P., Mahdhiya N.Z., Yoviana I., Jessica J., Komariah M. (2025). Early Mobilization in Post-Orthopedic Surgery Patients: A Scoping Review. J. Multidiscip. Healthc..

[6] Chan E.Y., Fransen M., Parker D.A., Assam P.N., Chua N. (2014). Femoral nerve blocks for acute postoperative pain after knee replacement surgery. Cochrane Database Syst. Rev..

[7] Wang D., Yang Y., Li Q., Tang S.-L., Zeng W.-N., Xu J., Xie T.-H., Pei F.-X., Yang L., Li L.-L. (2017). Adductor canal block versus femoral nerve block for total knee arthroplasty: A meta-analysis of randomized controlled trials. Sci. Rep..

[8] Fillingham Y.A., Hannon C.P., Kopp S.L., Austin M.S., Sershon R.A., Stronach B.M., Meneghini R.M., Abdel M.P., Griesemer M.E., Woznica A. (2022). The Efficacy and Safety of Regional Nerve Blocks in Total Knee Arthroplasty: Systematic Review and Direct Meta-Analysis. J. Arthroplast..

[9] Koepke E.J., Manning E.L., Miller T.E., Ganesh A., Williams D.G.A., Manning M.W. (2018). The rising tide of opioid use and abuse: The role of the anesthesiologist. Perioper. Med..

[10] Chen Q., Chen E., Qian X. (2021). A Narrative Review on Perioperative Pain Management Strategies in Enhanced Recovery Pathways—The Past, Present and Future. J. Clin. Med..

[11] Zhao C., Liao Q., Yang D., Yang M., Xu P. (2024). Advances in perioperative pain management for total knee arthroplasty: A review of multimodal analgesic approaches. J. Orthop. Surg. Res..

[12] Chou R., Gordon D.B., de Leon-Casasola O.A., Rosenberg J.M., Bickler S., Brennan T., Carter T., Cassidy C.L., Chittenden E.H., Degenhardt E. (2016). Management of Postoperative Pain: A Clinical Practice Guideline From the American Pain Society, the American Society of Regional Anesthesia and Pain Medicine, and the American Society of Anesthesiologists’ Committee on Regional Anesthesia, Executive Committee, and Administrative Council. J. Pain.

[13] Karam J.A., Schwenk E.S., Parvizi J. (2021). An Update on Multimodal Pain Management After Total Joint Arthroplasty. J. Bone Jt. Surg. Am..

[14] Nguyen L.C., Sing D.C., Bozic K.J. (2016). Preoperative Reduction of Opioid Use Before Total Joint Arthroplasty. J. Arthroplast..

[15] Colvin L.A., Bull F., Hales T.G. (2019). Perioperative opioid analgesia—When is enough too much? A review of opioid-induced tolerance and hyperalgesia. Lancet.

[16] Mazda Y., Peacock S., Wolfstadt J., Matelski J., Chan V., Gleicher Y.J. (2021). Developing a business case for a regional anesthesia block room: Up with efficiency, down with costs. Reg. Anesth. Pain Med..

[17] Carvalho B., Yun R.D., Mariano E.R. (2015). Continuous Versus Single-Injection Peripheral Nerve Blocks: A Prospective Cohort Study Comparing Procedural Time and Estimated Personnel Cost. Open Anesthesiol. J..

[18] Niyonkuru E., Iqbal M.A., Zhang X., Ma P. (2025). Complementary Approaches to Postoperative Pain Management: A Review of Non-pharmacological Interventions. Pain Ther..

[19] Motamed C., Mazoit X., Ghanouchi K., Guirimand F., Abhay K., Lieutaud T., Bensaid S., Fernandez C., Duvaldestin P. (2000). Preemptive Intravenous Mo~bine-6-glucuroni~e Is Ineffective for Postoperative Pain Relief. Anesthesiology.

[20] Wadhwa A., Clarke D., Goodchild C.S., Young D. (2001). Large-Dose Oral Dextromethorphan as an Adjunct to PatientControlled Analgesia with Morphine after Knee Surgery. Anesth. Analg..

[21] Zippel H., Wagenitz A. (2006). Comparison of the Efficacy and Safety of Intravenously Administered Dexketoprofen Trometamol and Ketoprofen in the Management of Pain after Orthopaedic Surgery. Clin. Drug Investig..

[22] Sivrikoz N., Koltka K., Guresti E., Buget M., Senturk M., Ozyalcin S. (2014). Perioperative dexketoprofen or lornoxicam administration for pain management after major orthopedic surgery: A randomized, controlled study. Agri.

[23] Chen H.J., Chen T.Y., Huang C.Y., Hsieh Y.M., Lai H.L. (2015). Effects of music on psychophysiological responses and opioid dosage in patients undergoing total knee replacement surgery. Jpn. J. Nurs. Sci..

[24] Ban W.R., Zhang E.A., Lv L.F., Dang X.Q., Zhang C. (2017). Effects of periarticular injection on analgesic effects and NSAID use in total knee arthroplasty and total hip arthroplasty. Clinics.

[25] Politi J.R., Davis R.L., Matrka A.K. (2017). Randomized Prospective Trial Comparing the Use of Intravenous versus Oral Acetaminophen in Total Joint Arthroplasty. J. Arthroplast..

[26] Hickman S.R., Mathieson K.M., Bradford L.M., Garman C.D., Gregg R.W., Lukens D.W. (2018). Randomized trial of oral versus intravenous acetaminophen for postoperative pain control. Am. J. Health Syst. Pharm..

[27] Xiao X., Zhang Q., Ouyang Z., Guo X. (2018). Comparison of perioperative flurbiprofen axetil or celecoxib administration for pain management after total-knee arthroplasty: A retrospective study. Medicine.

[28] Kim S.H., Kim D.H., Shin S., Kim S.J., Kim T.L., Choi Y.S. (2019). Effects of dexmedetomidine on inflammatory mediators after tourniquet-induced ischemia-reperfusion injury: A randomized, double-blinded, controlled study. Minerva Anestesiol..

[29] Shao Y., Zhao X., Zhai Y., Yang J., Wang S., Liu L., Wang J. (2020). Comparison of analgesic effect, knee joint function recovery, and safety profiles between pre-operative and post-operative administrations of meloxicam in knee osteoarthritis patients who underwent total knee arthroplasty. Ir. J. Med. Sci..

[30] Peng H., Wang W., Lin J., Weng X., Qian W., Wang W. (2021). Local Efficacy of Corticosteroids as an Adjuvant for Periarticular Cocktail Injection in Simultaneous Bilateral Total Knee Arthroplasty: A Prospective Randomized Double-Blind Controlled Trial. Pain Res. Manag..

[31] Coviello M., Abate A., Ippolito F., Nappi V., Maddalena R., Maccagnano G., Noia G., Caiaffa V. (2022). Continuous Cold Flow Device Following Total Knee Arthroplasty: Myths and Reality. Medicina.

[32] Yuan M., Tang T., Ding Z., Li H., Zhou Z. (2022). Analgesic effect of perioperative duloxetine in patients after total knee arthroplasty: A prospective, randomized, double-blind, placebo-controlled trial. BMC Musculoskelet. Disord..

[33] Paulin V.S., Bakshi S.G., Hegde P.C., Rathod A., Gulia A., Kulkarni A.M., Paramanandam V.S. (2023). Inkk Trial .Çô Intraoperative ketamine for perioperative pain management following total knee endoprosthetic replacement in oncology: A double-blinded randomized trial. Braz. J. Anesthesiol..

[34] Kraus M.B., Bingham J.S., Kekic A.P., Erickson C.B., Grilli C.B.P., Seamans D.P., Upjohn D.P., Hentz J.G., Clarke H.D., Spangehl M.J. (2024). Does Preoperative Pharmacogenomic Testing of Patients Undergoing TKA Improve Postoperative Pain? A Randomized Trial. Clin. Orthop. Relat. Res..

[35] Sağlam S., Karaduman Z.O., Arıcan M., Yücel M.O., Dalaslan R.E., Cangur S., Uludag V. (2025). The role of tranexamic acid and cryotherapy on acute postoperative pain and blood loss: A randomized controlled study following total knee arthroplasty. Eur. J. Orthop. Surg. Traumatol..

[36] Koinig H. (1998). Magnesium Sulfate Reduces Intra- and Postoperative Analgesic Requirements. Anesth. Analg..

[37] Joshi W., Reuben S.S., Kilaru P.R., Sklar J., Maciolek H. (1999). Postoperative Analgesia for Outpatient Arthroscopic Knee Surgery with Intraarticular Clonidine. Anesth. Analg..

[38] Gentili M., Enel D., Szymskiewicz O., Mansour F., Bonnet F. (2001). Postoperative analgesia by intraarticular clonidine and neostigmine in patients undergoing knee arthroscopy. Reg. Anesth. Pain Med..

[39] Vranken J.H., Vissers K.C., de Jongh R., Heylen R. (2001). Intraarticular Sufentanil Administration Facilitates Recovery After Day-Case Knee Arthroscopy. Anesth. Analg..

[40] Ménigaux C., Adam F., Guignard B., Sessler D.I., Chauvin M. (2005). Preoperative Gabapentin Decreases Anxiety and Improves Early Functional Recovery From Knee Surgery. Anesth. Analg..

[41] Arai Y.C., Ikeuchi M., Fukunaga K., Ueda W., Kimura T., Komatsu T. (2006). Intra-articular injection of warmed lidocaine improves intraoperative anaesthetic and postoperative analgesic conditions. Br. J. Anaesth..

[42] Jacobson E., Assareh H., Cannerfelt R., Renstrom P., Jakobsson J. (2006). Pain after elective arthroscopy of the knee: A prospective, randomised, study comparing conventional NSAID to coxib. Knee Surg. Sports Traumatol. Arthrosc..

[43] Tashjian R.Z., Banerjee R., Bradley M.P., Alford W., Fadale P.D. (2006). Zolpidem Reduces Postoperative Pain, Fatigue, and Narcotic Consumption Following Knee Arthroscopy: A Prospective Randomized Placebo-Controlled Double-Blinded Study. J Knee Surg..

[44] Hashemi S.J., Soltani H., Heidari S.M., Rezakohanfekr M. (2013). Preemptive analgesia with intra-articular pethidine reduces pain after arthroscopic knee surgery. Adv. Biomed. Res..

[45] Sun R., Zhao W., Hao Q., Tian H., Tian J., Li L., Jia W., Yang K. (2014). Intra-articular clonidine for post-operative analgesia following arthroscopic knee surgery: A systematic review and meta-analysis. Knee Surg. Sports Traumatol. Arthrosc..

[46] Isik C., Demirhan A., Yetis T., Okmen K., Sarman H., Tekelioglu U.Y., Duran T. (2015). Efficacy of intraarticular application of ketamine or ketamine-levobupivacaine combination on post-operative pain after arthroscopic meniscectomy. Knee Surg. Sports Traumatol. Arthrosc..

[47] Onda A., Ogoshi A., Itoh M., Nakagawa T., Kimura M. (2016). Comparison of the effects of treatment with celecoxib, loxoprofen, and acetaminophen on postoperative acute pain after arthroscopic knee surgery: A randomized, parallel-group trial. J. Orthop. Sci..

[48] Zhou F., Du Y., Huang W., Shan J., Xu G. (2017). The efficacy and safety of early initiation of preoperative analgesia with celecoxib in patients underwent arthroscopic knee surgery: A randomized, controlled study. Medicine.

[49] Uribe A.A., Arbona F.L., Flanigan D.C., Kaeding C.C., Palettas M., Bergese S.D. (2018). Comparing the Efficacy of IV Ibuprofen and Ketorolac in the Management of Postoperative Pain Following Arthroscopic Knee Surgery. A Randomized Double-Blind Active Comparator Pilot Study. Front. Surg..

[50] El Baz M.M., Farahat T.E.M. (2019). Efficacy of Adding Dexmedetomidine to Intra-articular Levobupivacaine on Postoperative Pain after Knee Arthroscopy. Anesth. Essays Res..

[51] Hou J., Li W., Chen Y., Yang L., Li L., Zhao L. (2019). Early preoperative versus postoperative administration of meloxicam in pain control, patient global status improvement, knee function recovery of arthroscopic knee surgery. Medicine.

[52] Sağır Ö., Tatar B., Ugün F., Demir H.F., Balkaya A.N., Meriç G., Kocaoğlu N., Köroğlu A. (2020). Effects of intraarticular ketamine combined with periarticular bupivacaine on postoperative pain after arthroscopic meniscectomy. Jt. Dis. Relat. Surg..

[53] Liu J., Di J., Zhang Y., Xing E. (2021). Oxycodone-paracetamol tablet exhibits increased analgesic efficacy for acute postoperative pain, higher satisfaction and comparable safety profiles compared with celecoxib in patients underwent arthroscopic knee surgery. Inflammopharmacology.

[54] Barber F.A., Gladu D. (1998). Comparison of Oral Ketorolac and Hydrocodone for Pain Relief After Anterior Cruciate Ligament Reconstruction. J. Arthrosc. Relat. Surg..

[55] Al-Metwalli R.R., Mowafi H.A., Ismail S.A., Siddiqui A.K., Al-Ghamdi A.M., Shafi M.A., El-Saleh A.-R. (2008). Effect of intra-articular dexmedetomidine on postoperative analgesia after arthroscopic knee surgery. Br. J. Anaesth..

[56] Armellin G., Nardacchione R., Ori C. (2008). Intra-articular sufentanil in multimodal analgesic management after outpatient arthroscopic anterior cruciate ligament reconstruction: A prospective, randomized, double-blinded study. Arthroscopy.

[57] Elseify Z.A., El-Khattab S.O., Khattab A.M., Atta E.M., Ajjoub L.F. (2011). Combined parecoxib and I.V. paracetamol provides additional analgesic effect with better postoperative satisfaction in patients undergoing anterior cruciate ligament reconstruction. Saudi J. Anaesth..

[58] Mardani-Kivi M., Mobarakeh M.K., Haghighi M., Naderi-Nabi B., Sedighi-Nejad A., Hashemi-Motlagh K., Saheb-Ekhtiari K. (2013). Celecoxib as a pre-emptive analgesia after arthroscopic knee surgery; a triple-blinded randomized controlled trial. Arch. Orthop. Trauma Surg..

[59] Ma L., Zhang L., Wang H., Jiang C. (2021). Efficiency and safety: Comparison between preoperative analgesia and postoperative analgesia using non-steroidal anti-inflammatory drugs in patients receiving arthroscopic knee surgery in a multicenter, randomized, controlled study. Inflammopharmacology.

[60] Wang C., Yang P., Zhang D., Jeon I.-H., Yu T., Zhang Y., Qi C. (2021). Effects of Temperature and Volume of Intraoperative Normal Saline Irrigation on Postoperative Pain, Swelling, and Serum Markers of Inflammation in Patients After Elective, Arthroscopic, Single-Bundle Surgical Reconstruction of the Anterior Cruciate Ligament: A Retrospective, Single-Center Study. Med. Sci. Monit..

[61] Alipour M., Attar A.S., Akbari A., Sheybani S., Ariamanesh A.S., Bakhtiari E., Khademi S.H., Makhmalbaf H., Farahi A. (2023). Intra-articular remifentanil on postoperative pain in knee arthroscopic surgery. J. Orthop. Sci..

[62] Doleman B., Leonardi-Bee J., Heinink T.P., Boyd-Carson H., Carrick L., Mandalia R., Lund J.N., Williams J.P. (2021). Pre-emptive and preventive NSAIDs for postoperative pain in adults undergoing all types of surgery. Cochrane Database Syst. Rev..

[63] Lu B., Tian A.-X., Fan Z.-R., Zhao X.-W., Jin H.-Z., Ma J.-X., Ma X.-L. (2025). Effectiveness of oral vs intravenous acetaminophen on pain management following total joint arthroplasty: A systematic review and meta-analysis. World J. Orthop..

[64] Botti M., Kent B., Bucknall T., Duke M., Johnstone M.-J., Considine J., Redley B., Hunter S., de Steiger R., Holcombe M. (2014). Development of a Management Algorithm for Post-operative Pain (MAPP) after total knee and total hip replacement: Study rationale and design. Implement. Sci..

[65] Ferreira G.E., Patanwala A.E., Turton H., Langford A.V., Harris I.A., Maher C.G., McLachlan A.J., Glare P., Lin C.-W.C. (2024). How is postoperative pain after hip and knee replacement managed? An analysis of two large hospitals in Australia. Perioper. Med..

[66] Pirri C., Sorbino A., Manocchio N., Pirri N., Devito A., Foti C., Migliore A. (2024). Chondrotoxicity of Intra-Articular Injection Treatment: A Scoping Review. Int. J. Mol. Sci..

[67] Gelman D., Gelmanas A., Urbanaitė D., Tamošiūnas R., Sadauskas S., Bilskienė D., Naudžiūnas A., Širvinskas E., Benetis R., Macas A. (2018). Role of Multimodal Analgesia in the Evolving Enhanced Recovery after Surgery Pathways. Medicina.

[68] Cheung C.K., Adeola J.O., Beutler S.S., Urman R.D. (2022). Postoperative Pain Management in Enhanced Recovery Pathways. J. Pain Res..

[69] Fan M., Chen Z. (2020). A systematic review of non-pharmacological interventions used for pain relief after orthopedic surgical procedures. Exp. Ther. Med..

[70] Fan Chiang Y.H., Wang M.T., Chan S.M., Chen S.Y., Wang M.L., Hou J.D., Tsai H.C., Lin J.A. (2023). Motor-Sparing Effect of Adductor Canal Block for Knee Analgesia: An Updated Review and a Subgroup Analysis of Randomized Controlled Trials Based on a Corrected Classification System. Healthcare.

[71] Darville-Beneby R., Lomanowska A.M., Yu H.C., Jobin P., Rosenbloom B.N., Gabriel G., Daudt H., Negraeff M., Di Renna T., Hudspith M. (2023). The Impact of Preoperative Patient Education on Postoperative Pain, Opioid Use, and Psychological Outcomes: A Narrative Review. Can. J. Pain.

[72] Bellomo R., Warrillow S.J., Reade M.C. (2009). Why we should be wary of single-center trials. Crit. Care Med..

[73] Gheorghe A., Roberts T.E., Ives J.C., Fletcher B.R., Calvert M. (2013). Centre selection for clinical trials and the generalisability of results: A mixed methods study. PLoS ONE.

[74] Naik B.I., Kuck K., Saager L., Kheterpal S., Domino K.B., Posner K.L., Sinha A., Stuart A., Brummett C.M., Durieux M.E. (2022). Practice Patterns and Variability in Intraoperative Opioid Utilization: A Report From the Multicenter Perioperative Outcomes Group. Anesth. Analg..

