# Supplementary material for: Postoperative Pain Management Strategies Without Regional Analgesia in Knee Surgeries: A Scoping Review
Source: Med Sci (Basel). 2026 Jan 30;14(1):62. doi: 10.3390/medsci14010062 (PMC12921793; doi:10.3390/medsci14010062)
Supplement: Supplementary file 1 [file medsci-14-00062-s001.zip › Supplementary material 1.pdf]

Search strategy

Scoping review of literature for postoperative pain management strategies without regional analgesia in knee surgeries.

Concept: 1 postoperative pain management

Concept: 2 knee surgeries

Concept: 3 no regional analgesia

Postoperative pain management	Knee surgeries	Regional analgesia
Pain	• Knee surgery<br>• Knee operation<br>• Knee procedure	• Regional analgesia<br>• Regional anesthesia<br>• Peripheral nerve block(s)<br>• Nerve block(s)<br>• Local anesthesia<br>• Local analgesia
nociception	• Total knee arthroplasty (TKA)<br>• Partial knee arthroplasty (PKA)<br>• Unicompartmental knee arthroplasty (UKA)<br>• Revision knee arthroplasty	• Femoral nerve block<br>• Sciatic nerve block<br>• Popliteal nerve block<br>• Adductor canal block (saphenous nerve block)<br>• Lateral femoral cutaneous nerve block<br>• Obturator nerve block
discomfort	• Knee arthroscopy<br>• Diagnostic knee arthroscopy<br>• Therapeutic knee arthroscopy	• Continuous peripheral nerve block<br>• Single-shot nerve block<br>• Ultrasound-guided nerve block<br>• Catheter-based nerve block<br>• Nerve stimulator
• Ache<br>• Soreness<br>• Distress<br>• Tenderness	• Anterior cruciate ligament reconstruction (ACL reconstruction)<br>• Posterior cruciate ligament reconstruction (PCL reconstruction)<br>• Multiligament knee reconstruction	
	• Patellar realignment<br>• Patellar tendon repair	
• Pain<br>• Postoperative pain<br>• Acute pain<br>• Chronic pain<br>• Nociceptive pain<br>• Neuropathic pain<br>• Pain management	• Tibial plateau fracture fixation<br>• Distal femoral fracture fixation	
• Algia	• Knee replacement surgery	

• Nociception<br>• Hyperalgesia<br>• Allodynia<br>• Dysesthesia<br>• Neuralgia	• Knee ligament repair<br>• Surgical management of knee injuries<br>• Minimally invasive knee surgery	
	• Recovery after knee surgery<br>• Pain management after knee surgery<br>• Rehabilitation after knee surgery<br>• Functional outcomes<br>• Range of motion	PUBMED<br>("regional analgesia" OR "regional anesthesia" OR "nerve block" OR "peripheral nerve block" OR "local anesthesia") AND<br>("pain management" OR "postoperative pain" OR "acute pain") AND<br>("femoral nerve block" OR "sciatic nerve block" OR "adductor canal block" OR "interscalene block")
	("knee surgery" OR "knee arthroplasty" OR "knee arthroscopy" OR "anterior cruciate ligament reconstruction" OR "meniscus repair" OR "cartilage repair") AND ("postoperative pain" OR "pain management" OR "rehabilitation" OR "functional recovery")	EMBASE<br>('regional analgesia' OR 'nerve block' OR 'peripheral nerve block') AND<br>('pain management' OR 'postoperative pain') AND<br>('femoral nerve block' OR 'sciatic nerve block' OR 'adductor canal block')

SCOPUS

(TITLE-ABS-KEY (pain AND management AND postoperative AND knee AND surgery) AND NOT (nerve AND block OR block OR "peripheral nerve block" OR bupivacaine OR ropivacaine OR spinal AND anaesthesia OR epidural AND anaesthesia) AND PUBYEAR > 2014 AND PUBYEAR < 2025) AND (LIMIT-TO (SUBJAREA , "MEDI")) AND (LIMIT-TO (DOCTYPE , "ar")) AND (LIMIT-TO (LANGUAGE , "English"))

EMBASE

#	Query	Results from 26 Jan 2025
1	exp pain/	1,920,035
2	postoperative.mp. [mp=title, abstract, heading word, drug trade name, original title, device manufacturer, drug manufacturer, device trade name, keyword heading word, floating subheading word, candidate term word]	1,421,460
3	exp knee/	112,446
4	exp surgery/	6,907,767

5	(arthroplasty or arthroscopy or replacement or substitution).mp. [mp=title, abstract, heading word, drug trade name, original title, device manufacturer, drug manufacturer, device trade name, keyword heading word, floating subheading word, candidate term word]	1,046,260
6	1 and 2 and 3 and 4 and 5	1,256

Clinicaltrials.gov

Showing results for: knee I Other terms: Pain Management I Surgery I Completed studies I Adult (18 - 64), Older adult (65+)

Revised criteria

Population:

- Adults (≥18 years) undergoing total knee arthroplasty (TKA) or hemi knee replacement .All types of knee surgeries i.e. arthroscopies, ligament repair etc.

Interventions:

- Any postoperative pain management strategy that does not hinder early mobilization or same-day discharge.
- Includes systemic pharmacological regimens (opioids, NSAIDs/COX-2 inhibitors, acetaminophen, gabapentinoids, NMDA antagonists, corticosteroids, alpha-2 agonists, nefopam, magnesium, duloxetine, ketamine, IV lidocaine).
- Includes local infiltration analgesia (LIA) with local anesthetic ± adjuncts if no indwelling catheter is required.
- Includes intra-articular or peri-articular non-local-anesthetic agents (e.g., morphine, ketorolac, corticosteroids).
- Includes non-pharmacological interventions (e.g., cryotherapy, TENS) if used postoperatively and compatible with mobilization.

Comparators:

- Placebo, usual care, or another eligible non-mobility-limiting analgesic regimen.

Outcomes:

- Reports acute postoperative pain (VAS/NRS/VRS) ≤48–72 hours, and/or measures of early mobilization, length of stay (LOS), or discharge rate.
- At least one outcome relevant to early discharge (pain scores, opioid consumption, mobilization time/distance, readiness-for-discharge score, LOS).

Study types:

- RCTs, non-RCTs, prospective and retrospective comparative studies.

Revised Exclusion Criteria

- Anesthetic/analgesic techniques likely to delay mobilization:
    - Spinal or epidural analgesia with indwelling catheter.
    - Spinal or epidural anesthesia/analgesia with intrathecal opioid.
    - Peripheral nerve blocks that produce motor weakness or require catheter: femoral nerve block, adductor canal block, sciatic nerve block, saphenous block, iPACK block if motor involvement reported.
- Continuous wound/nerve catheters of any kind.
- Intra-articular local anesthetics (lignocaine, bupivacaine, ropivacaine) as the main intervention (short duration, not aligned with early discharge aims).
- Surgical technique studies, ERAS pathway studies where analgesia cannot be separated from other interventions.
- Long-term pain studies only (>3 months).
