# Supplementary material for: Postoperative Pain Management Strategies Without Regional Analgesia in Knee Surgeries: A Scoping Review
Source: Med Sci (Basel). 2026 Jan 30;14(1):62. doi: 10.3390/medsci14010062 (PMC12921793; doi:10.3390/medsci14010062)

Figures and tables

1. Countries of origin

Country/Region	Frequency
China	9
USA	6
France	3
Turkey	3
Italy	2
Iran	2
Japan	2
Egypt	2
Belgium	2
Germany	1
Austria	1
Australia	1
Sweden	1
Saudi Arabia	1
South Africa	1
Iran	1
Qatar	1
Turkey	1
India	1
South Korea	1
Taiwan	1
Netherlands	1

2. Study designs

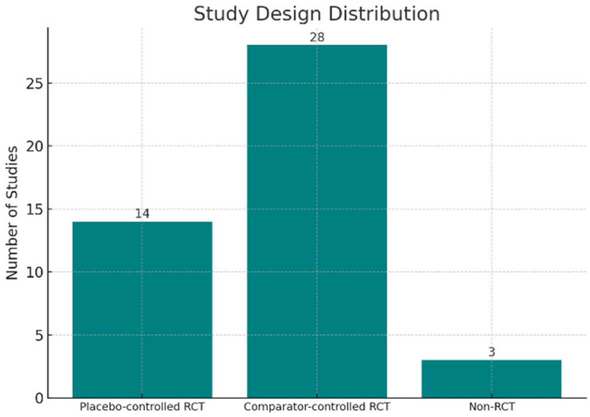

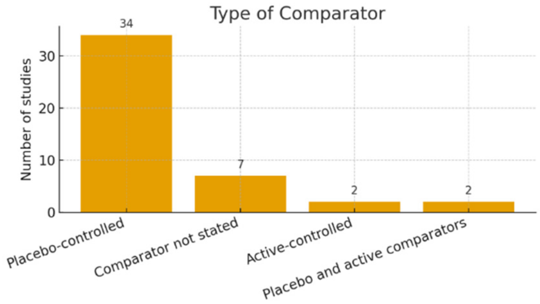

3. Demographics

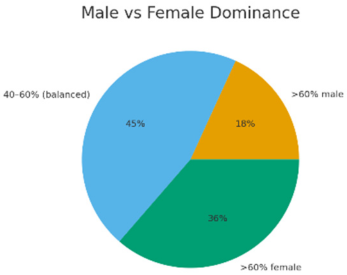

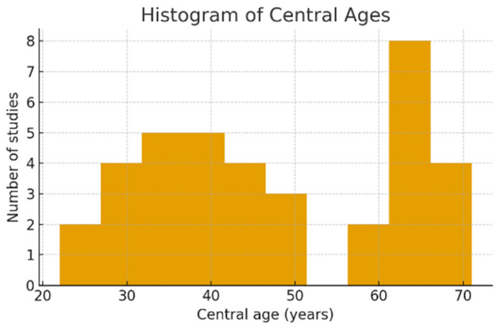

4. Types of surgery

Type of Surgery	Frequency
CLMR	08
TKR	17
ASCOPY	19

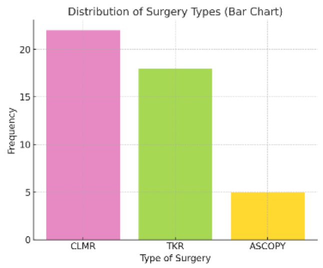

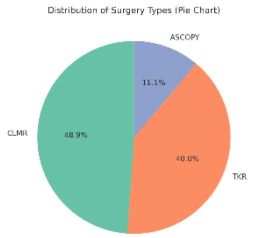

Surgery Category (Detailed)	Number of Studies
Unilateral TKA/TKR	10
Arthroscopic knee surgery (mixed)	10
Arthroscopic meniscectomy	7
Arthroscopic ACL reconstruction	6
Mixed THA+TKA (hip + knee)	3
Bilateral TKA	2
ACL reconstruction (unspecified approach)	2
Open knee surgery (non-arthroscopy)	2
Oncologic knee endoprosthesis	1
Arthroscopic meniscus repair	1
Diagnostic knee arthroscopy	1

5. Type of analgesic intervention

Category	Number of Studies

Intra-articular	11
Periarticular	4
Systemic (IV/Oral)	23
Combined	3
Non-pharmacological	8

6. Pain scale distribution

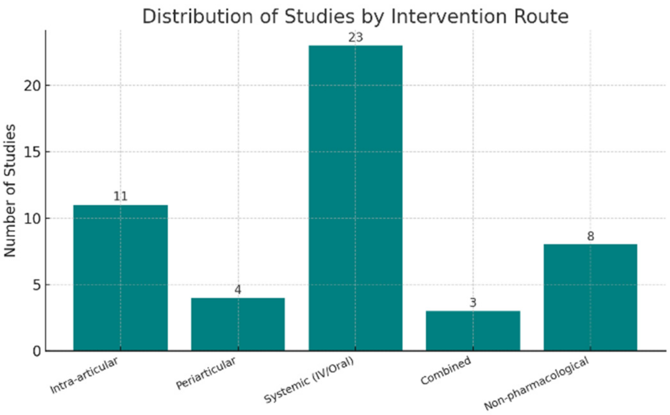

Pain Scale	Number of Studies
VAS 0–10	25
VAS 0–100 mm	16
NRS 0–10 (incl. VNRS)	3
Categorical pain scale	1
Other pain-related scales (OBAS, SPID/TOTPAR, PGA)	3

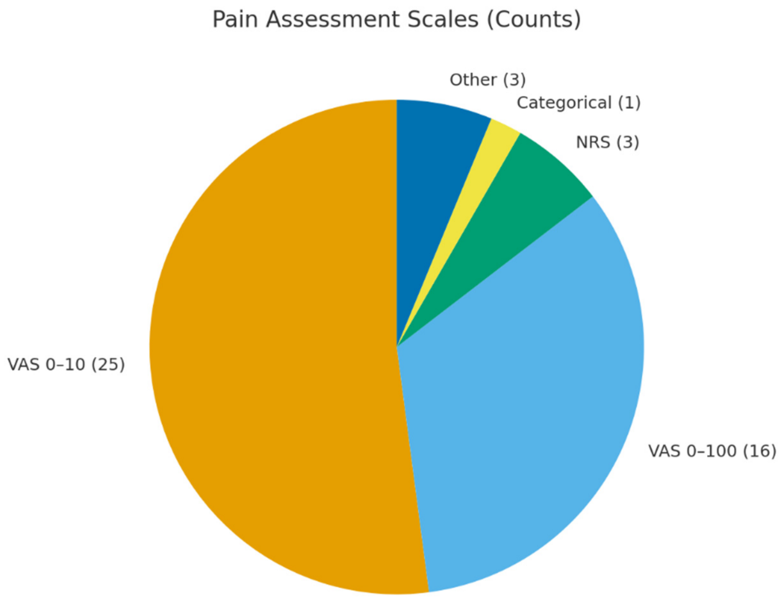
Pain Assessment Scales (Counts)
Other (3)
Categorical (1)
NRS (3)
VAS 0–10 (25)
VAS 0–100 (16)

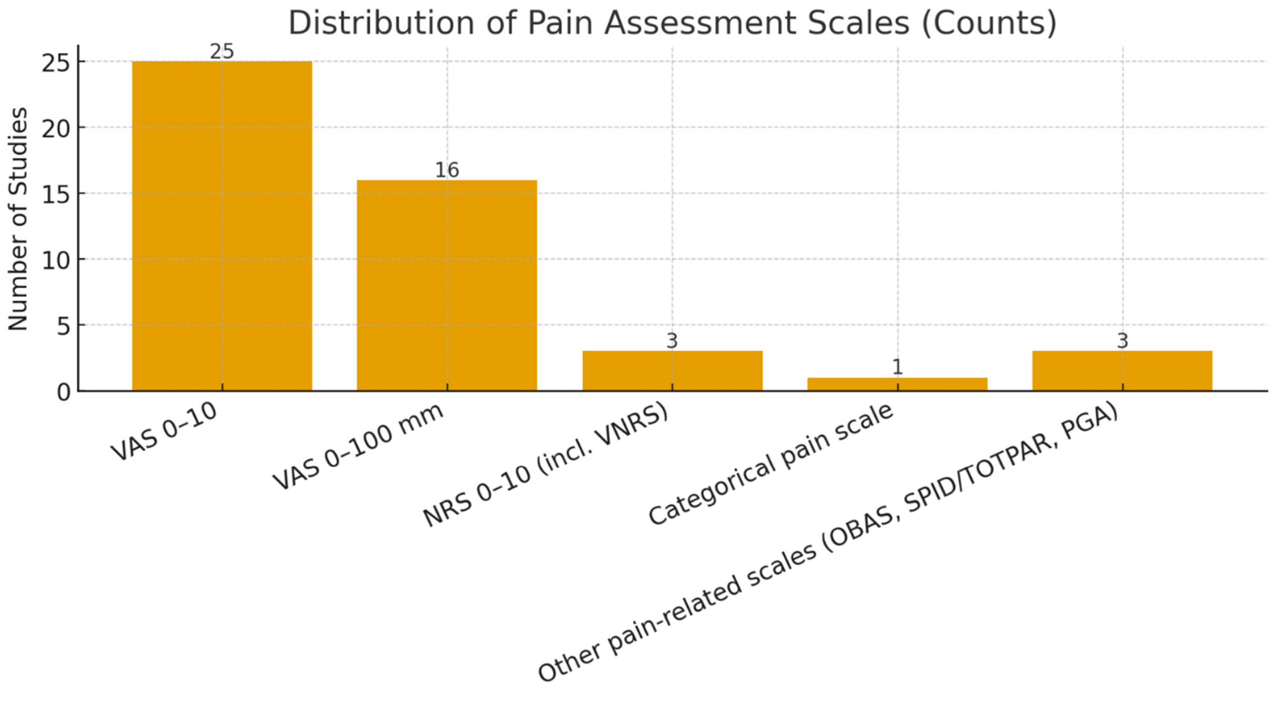
Distribution of Pain Assessment Scales (Counts)
Number of Studies
0
5
10
15
20
25
25
16
3
1
3
VAS 0–10
VAS 0–100 mm
NRS 0–10 (incl. VNRS)
Categorical pain scale
Other pain-related scales (OBAS, SPID/TOTPAR, PGA)

7. Outcome distribution: pain, mobilization and length-of-stay (LOS)

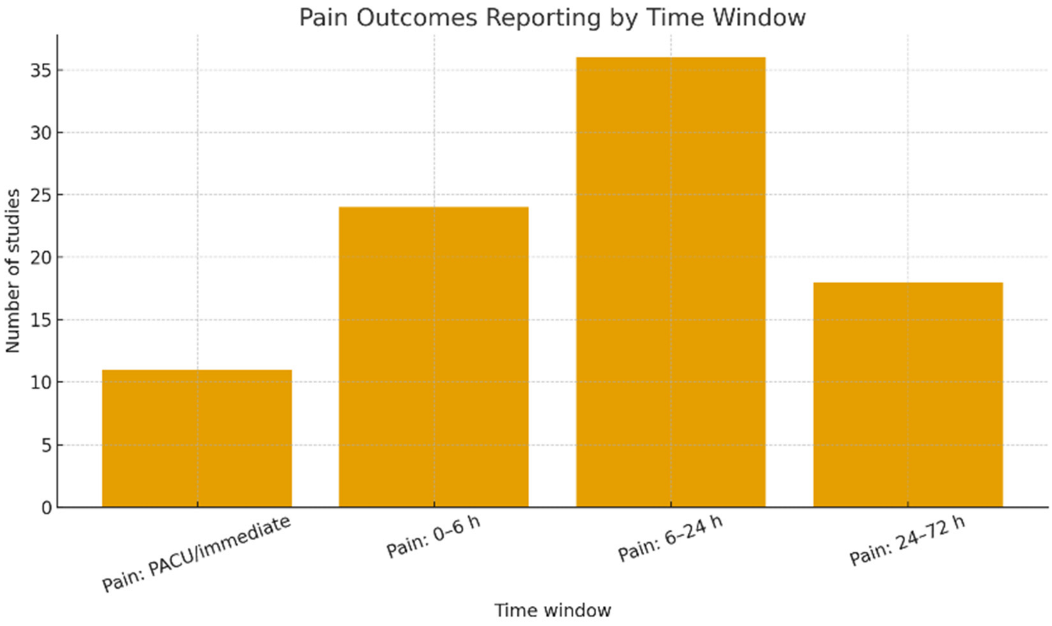

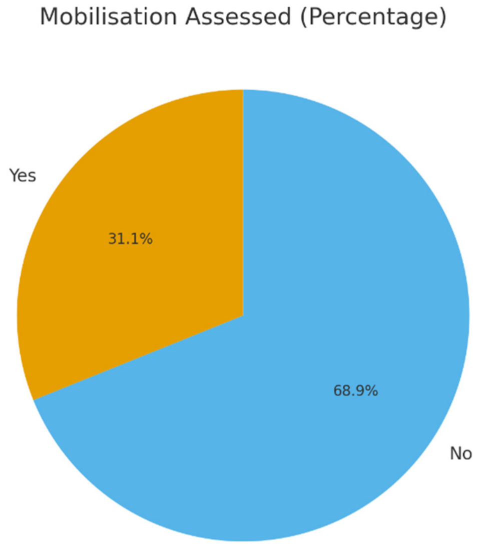

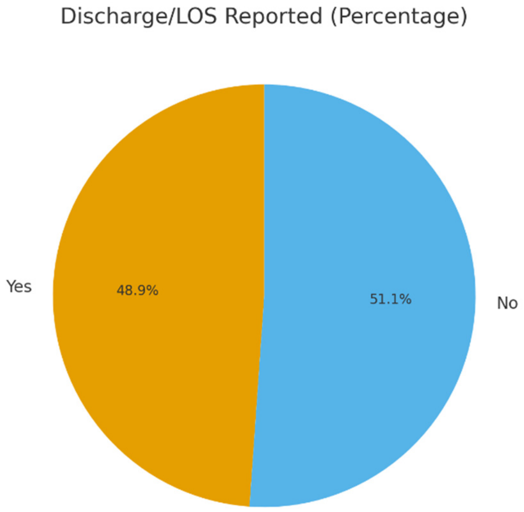

Supplement: Supplementary file 1 [file medsci-14-00062-s001.zip › Supplementary material S2.pdf]
